# Gallbladder carcinosarcoma with two heterologous components: a case report

**DOI:** 10.11604/pamj.2022.42.284.36311

**Published:** 2022-08-16

**Authors:** El Mehdi Tiabi, Achraf Miry, Anas Haloui, Mohammed Bouziane, Imane Skiker, Nassira Karich, Amal Bennani

**Affiliations:** 1 Pathology Department, Mohammed VI University Hospital, Faculty of Medicine and Pharmacy of Oujda, Mohammed First University of Oujda, Oujda, Morocco,; 2 General Surgery Department, Mohammed VI University Hospital, Faculty of Medicine and Pharmacy of Oujda, Mohammed First University of Oujda, Oujda, Morocco,; 3 Radiology Department, Mohammed VI University Hospital, Faculty of Medicine and Pharmacy of Oujda, Mohammed First University of Oujda, Oujda, Morocco

**Keywords:** Carcinosarcoma, gallbladder, multidisciplinary therapy, case report

## Abstract

Carcinosarcoma of the gallbladder is a rare cancer characterized by presence of a carcinomatous and a sarcomatous component. In our work, we report the case of a 66-year-old male patient, presenting with isolated abdominal pain evolving for more than 6 months. contrast-enhanced computed tomography enabled identification of a gallbladder mass, invading liver, duodenum and abdominal wall. A cholecystectomy, extended to liver, duodenum and abdominal wall was performed. The final diagnosis of gallbladder carcinosarcoma was obtained by pathological assessment. Gallbladder carcinosarcoma has a poor prognosis. Since it is rare, no established chemotherapy or radiation protocols exist. Further studies about case series are needed to establish better therapeutic protocols. Gallbladder carcinosarcoma is a rare cancer with a rapid progression making therapeutic decisions difficult. All these factors contribute to the poor prognosis of this cancer.

## Introduction

Carcinosarcoma is a relatively rare gallbladder cancer (1% of all gallbladder cancers), with only about 100 reported cases in the English literature [1]. Histologically, this tumor is characterized by presence of two components: a carcinomatous component and a sarcomatous component [1]. Radical resection seems to be the only curative treatment, whereas adjuvant therapy is not effective.

Gallbladder carcinosarcoma (GBCS) has a poor prognosis, worse or similar to that of gallbladder adenocarcinoma [2]. In our work, we report the case of a locally advanced gallbladder carcinosarcoma in a 66-year-old man. The patient has undergone curative surgical resection. Follow-up of the patient with routine imaging and laboratory studies has shown appearance of hepatic metastases and patient´s death 4 years after resection.

## Patient and observation

**Patient information:** we report the case of a 66-year-old male patient, with a history of peritonitis secondary to gastric ulcer perforation 36 years ago. He presented for isolated abdominal pain on the right hypochondriac region evolving for more than 6 months. No jaundice was reported.

**Clinical findings:** physical examination was unremarkable, showing stable vital signs and normal abdominal examination: no mass was identified through clinical examination.

**Diagnostic assessment:** biological explorations showed marked elevation of total bilirubin (10.1 mg/dL), of alanine aminotransferase (ALAT) and aspartate aminotransferase (ASAT) with values of 224IU/L and 124 IU/L respectively and of alkaline phosphatase (880 IU/L), serum carcinoembryonic antigen level (CEA) was elevated. Other biological abnormalities include a biological inflammatory syndrome with elevated C reactive protein (CRP) at 85mg/L and anemia with a hemoglobin value at 15 μg/L. The patient underwent a contrast-enhanced computed tomography (CE-CT), which showed a large enhancing mass filling the hole gallbladder lumen, with extension to adjacent organs: liver, duodenum and abdominal wall.

**Figure 1:**
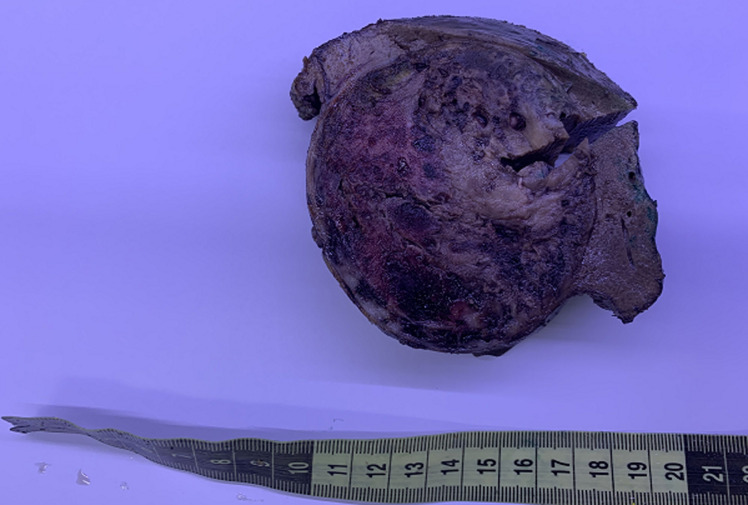
macroscopic photo of the tumor occupying the hole gallbladder and invading the hepatic parenchyma, the cut section shows a heterogenous appearance with presence of many hemorrhagic and necrotic foci

**Figure 2:**
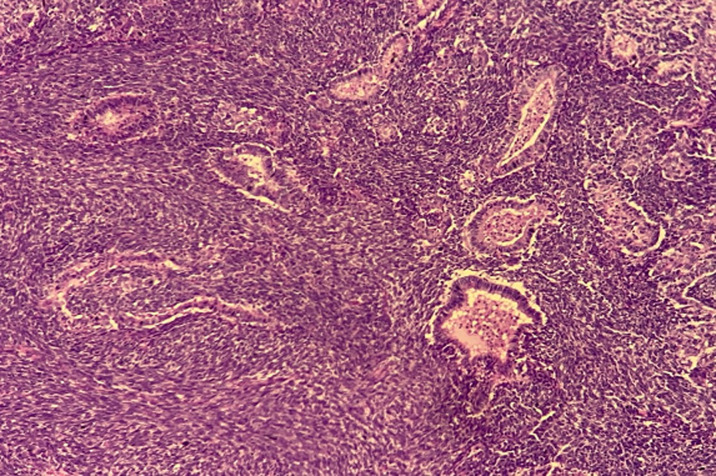
photomicroph of the carcinomatous component, made of irregular infiltrating tubes, made of markedly atypical large cells, HE; 100X

**Figure 3:**
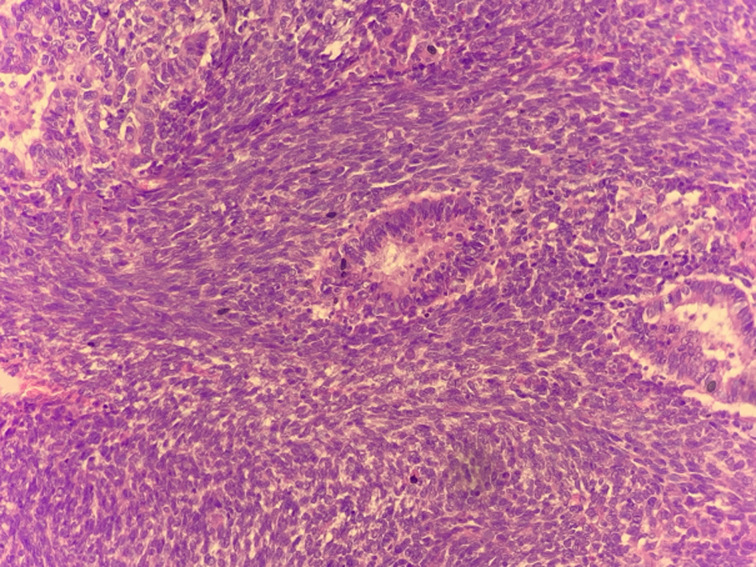
photomicrograph showing the sarcomatous component, made in this field of markedly atypical spindle cells, few tubes of the epithelial component can also be seen

**Figure 4:**
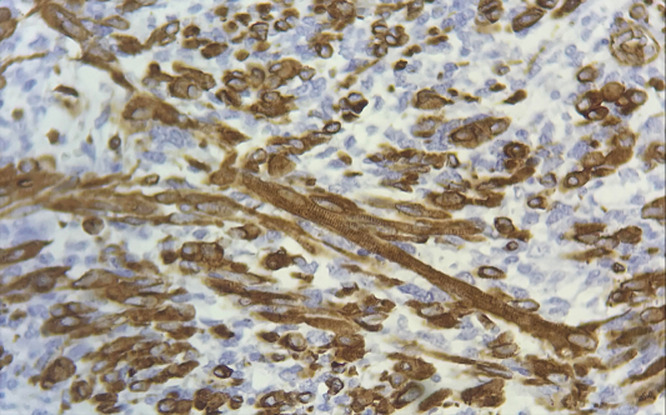
photomicrograph showing presence of cytoplasmic double striations, observed on the rhabdomyosarcomatous heterologous component of the sarcomatous component

**Figure 5:**
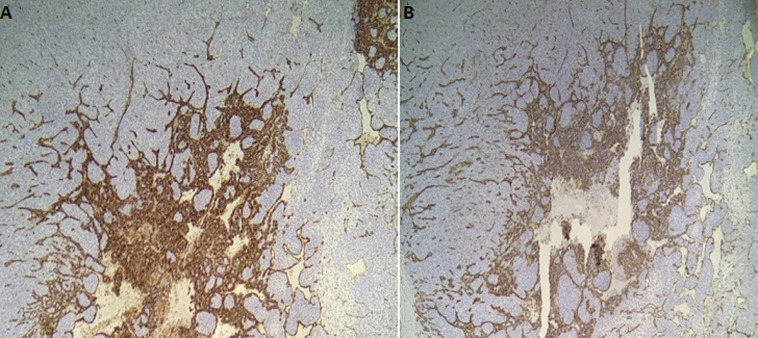
(A,B) photomicrograph showing expression of CD31 (left) and CD34 (right) by cells layering the anastomosing channels

**Therapeutic interventions:** the patient underwent a cholecystectomy, extended to liver, duodenum and to invaded abdominal wall. Macroscopic examination showed a tissular friable large, 15 x 8 x 6 cm mass filling the hole gallbladder lumen. Extension was identified towards the duodenal wall, the abdominal wall and to the liver. Cut section of the mass showed a heterogenous appearance with many diffuse necrotic and hemorrhagic foci (Figure 1). Microscopic examination showed a highly malignant neoplastic proliferation with two components: an epithelial, adenocarcinomatous component made of irregular infiltrating tubes, made of markedly atypical cells with large eosinophilic cytoplasm and large nuclei containing prominent nucleoli and showing frequent mitoses (Figure 2). This epithelial component showed cytoplasmic strong expression of cytokeratin 7. This antibody also enables identification of foci of epithelial component in which small clusters and isolated epithelial cells were present rather than real tubes. The sarcomatous component was mainly made of markedly atypical spindle cells. Nuclei were hyperchromatic and many mitoses could be observed (Figure 3). Two heterologous components could be identified in our case: a rhabdomyosarcomatous component made of elongated large cells with no morphologically visible double striations. These could be identified after use of the anti-desmin antibody (Figure 4). The second heterologous component was of vascular nature, showing foci of numerous anastomosing vascular channels, layered by squamous to cuboidal atypical cells with eosinophilic cytoplasm. The vascular nature could be confirmed after identifying expression of CD31 and CD34 by neoplastic cells (Figure 5).

**Diagnosis:** these morphological and immunohistochemical observations were consistent with a gallbladder carcinosarcoma. The tumor was invading the duodenal wall and the cystic canal resection margin with neoplastic margins. Extension to liver and abdominal wall were also confirmed microscopically.

**Follow-up and outcome of interventions:** follow-up of the patient with routine imaging and laboratory studies has shown appearance of hepatic metastases and patient´s death 4 years after resection.

**Informed consent:** the patient did give informed consent.

## Discussion

Among all tumors of the biliary tract, cancer of the gallbladder is the most frequent. However, carcinosarcomas, characterized by a combination of both carcinomatous and sarcomatous components, are relatively rare. There are only about 100 reported cases in the English literature [3]. The first reported case was in 1907 by Landsteiner *et al*. [3]. A male predilection is observed in cases of gallbladder carcinosarcoma (GBCS) with a sex ratio at 3,25: 1 [4]. However, in a large literature review about 78 GBCS cases, 72.4% of patients were female patients [5]. The affected patients have an average age of 68.8 years [4]. Pathogenesis of carcinosarcomas is not fully understood, and two types of carcinosarcomas can be identified: occurrence of collision between separate sarcoma and carcinoma in the gallbladder is called a true carcinosarcoma whereas occurrence of a sarcomatous differentiation in a poorly differentiated carcinoma is called the “so-called carcinosarcoma” [6]. In this latter category, the sarcomatous differentiation leads to morphological and immunohistochemical loss of any epithelial features on the sarcomatous part of the proliferation [6]. This latter category can also be called: a sarcomatoid or a spindle cell carcinoma [7].

In our case, the epithelial markers were expressed in the carcinomatous part and not in the sarcomatous part. The sarcomatous component may present in different appearances, with undifferentiated spindle cell proliferation being the most frequent one. The occurrence of a heterologuous component in form of rhabdomyosarcomatous component, as in our case, osteosarcomatous, chondrosarcomatous or a leiomyosarcomatous component have been reported in the literature [8]. The epithelial component is an adenocarcinoma in 79.2% of GBCS cases or a squamous cell carcinoma in the remaining 9.4% of cases [4]. A rare phenomenon would be presence of neuroendocrine differentiation, which was described in only 2 cases of GBCS [9,10].

Clinical features are not specific, and include, as for other gallbladder cancer types, anorexia, vomiting, abdominal pain and jaundice [10]. Abdominal pain seems to be the most frequently reported symptom (76.3% of cases) [5]. Only rare cases are reported to present no symptoms with a percentage of 2.6% according to a literature review about 78 cases [5]. Biologically, non-specific epithelial tumors markers can be elevated and include CEA, CA19-9 are not specific. Other biological explorations may show alteration of liver function test, reported in 43.9% of cases [5]. Imaging may show a papillary lesion projecting in form of papillae into the gallbladder lumen. Calcification can also be identified through imaging [10]. The tumor was located in the fundus in 34.9% of cases, in the body in 23.3% of cases and in the neck in 11.6% of cases [5].

Imaging may only help to categorize the observed lesion as a malignant lesion without being able to guide diagnosis toward a carcinosarcomatous lesion. In the literature, and as in our case, no case of GBCS has been diagnosed preoperatively. There is no consensus regarding the treatment of GBCS, although few reports about use of chemotherapy have been published [7]. Most cases who have undergone a neo-adjuvant chemotherapy have shown recurrences because of the presence of hepatic metastases [10]. The use of radiotherapy has shown no effectiveness. In the literature, radiotherapy have been shown to have effect only on the epithelial component with no effect on the sarcomatous component [10]. The only curative treatment remains surgery. Since the majority of cases of GBCS are diagnosed in an advances stage, with invasion of adjacent organs, the surgical treatment would be based on extended cholecystectomy [2].

Other types of gallbladder resection can be theoretically efficient, depending on extension of the carcinosarcoma. These include simple cholecystectomy or extended cholecystectomy including liver bed resection and pancreaticoduodenectomy. In terms of prognosis, and even with an aggressive surgical treatment, the prognosis and survival of GBCS cases remain poor. A median survival of 7 months with a 3-year survival rate of 31% has been reported by Okabayashi *et al*. [2]. In a similar study by Zhang *et al*. a median survival of 5 months with a 3-year survival rate of 16% has been reported [4]. In the literature, some factors have been reported to influence the prognosis, such as presence of infiltration, not exceeding the muscularis propria as being a good prognostic factor. A maximum diameter not exceeding 5cm has been described as a good prognostic factor, by Zhang *et al*. [4].

## Conclusion

Although gallbladder carcinosarcoma is a relatively rare neoplasm, it is certainly a very aggressive neoplasm, with usually a rapid progression. More studies are needed to identify different prognostic factors related to this highly malignant neoplasm, and to establish a consensus about therapeutic management. In our work, we report the case of a locally advanced gallbladder carcinosarcoma in a 66-year-old man. The patient has undergone curative surgical resection. Follow-up of the patient with routine imaging and laboratory studies has shown the appearance of hepatic metastases and patient´s death 4 years after resection.
